# A neural signature of the unique hues

**DOI:** 10.1038/srep42364

**Published:** 2017-02-10

**Authors:** Lewis Forder, Jenny Bosten, Xun He, Anna Franklin

**Affiliations:** 1 The Sussex Colour Group, University of Sussex, Falmer, BN1 9QH, United Kingdom; 2 School of Psychology, University of Sussex, Falmer, BN1 9QH, United Kingdom; 3 Cognition and Cognitive Neuroscience Research Centre, Department of Psychology, Bournemouth University, Poole, BH12 5BB, UK

## Abstract

Since at least the 17^th^ century there has been the idea that there are four simple and perceptually pure “unique” hues: red, yellow, green, and blue, and that all other hues are perceived as mixtures of these four hues. However, sustained scientific investigation has not yet provided solid evidence for a neural representation that separates the unique hues from other colors. We measured event-related potentials elicited from unique hues and the ‘intermediate’ hues in between them. We find a neural signature of the unique hues 230 ms after stimulus onset at a post-perceptual stage of visual processing. Specifically, the posterior P2 component over the parieto-occipital lobe peaked significantly earlier for the unique than for the intermediate hues (*Z* = −2.9, *p* = 0.004). Having identified a neural marker for unique hues, fundamental questions about the contribution of neural hardwiring, language and environment to the unique hues can now be addressed.

For more than five hundred years there has been the idea that there are four phenomenologically simple and pure “unique” hues^1^,^2^,^3^: red, yellow, green, and blue. It is claimed that unique hues are elemental qualities of color appearance because they cannot be described in terms of any other hues^4^, and that all other hues can be described as mixtures of the unique hues^5^. This concept of four unique hues can be traced in the history of both art and ancient architecture^1^,^2^. For example, in the 17^th^ century, Leonardo da Vinci stated that nature contains six simple colors: the unique hues of red, yellow, green and blue, plus black and white^1^,^6^. Unique hues are also central to classical theories of color vision such as Hering’s ‘opponent process theory’^3^ and modern models of color appearance^6^,^7^.

The existence of unique hues has been described as ‘one of the central mysteries of color science’^8^, and is also relevant to a broad range of fundamental debates in psychology, neuroscience, linguistics and philosophy. Color, and in particular color categories, have provided a key case for testing the opposing claims of Whorfians relativists^9^,^10^,^11^ and universalists^12^,^13^,^14^ over the causal direction of the relationship between language and cognition.

In neuroscience the unique hues have provided a key example for considering the link between neural activity and representation and subjective, conscious experience^6^,^8^. For philosophers, the monoadic irreducible nature of the qualia that correspond to the unique hues have provided a central focus for debates around the origin and nature of conscious experience^15^,^16^,^17^.

Results from cross-cultural studies suggest that color categories have a degree of universality, showing conservation across different color lexicons^13^, and whether the unique hues are the source of this universality has been debated^18^,^19^. The cross-cultural results on the universality of color categories and the assumed universality of phenomenological color experience have led to the assumption that there is a ‘hardwired’, neural representation of the unique hues somewhere in the visual system^20^. It was once thought that the unique hues must be represented at an early stage in the visual processing system, in the retina and lateral geniculate nucleus^21^. Since we now know that the retino-geniculate “opponent processes” are tuned to the intermediate color directions violet-chartreuse and cherry-teal, rather than to the unique hues^22^,^23^, models for the combination of retino-geniculate color mechanisms that result in a cortical color representation of the unique hues have been proposed^6^,^24^,^25^.

Only one study to date has reported evidence of a neural representation of unique hues: Stoughton and Conway^20^ took single-unit recordings in macaques and found that neurons in macaque posterior inferior temporal cortex (PIT) are preferentially tuned to unique red, green and blue, though not to unique yellow. However, Mollon^26^ challenged Stoughton and Conway’s conclusions arguing that their stimuli would be expected to maximally excite color-sensitive cells in the lateral geniculate nucleus, and that since PIT neurons could plausibly inherit “cardinal” geniculate color representations, their recordings do not convincingly demonstrate sensitivity of PIT neurons to the unique hues. Mollon advocated the need for a set of color stimuli that lie on a circle in an appropriate chromaticity space. Recently, Bohon *et al*.^27^ addressed this issue by taking electrophysiological recordings in macaque PIT to stimuli defined in a perceptually uniform color space. They found that activity in color-sensitive cells in PIT were best accounted for by simulated neurons that represent most directions in color space rather than the cardinal color directions favored in the lateral geniculate nucleus or the unique hues. They raised the possibility that variations in color saturation may have caused the strong neural responses to the unique hues reported in Soughton and Conway^20^. In short, identifying a neural representation of the unique hues has proved elusive, and scientists have questioned whether such a representation will be found^28^,^29^,^30^,^31^. Finding neural evidence for the unique hues remains a key objective in contemporary neuroscience.

The current study aimed to reveal a neural representation of the unique hues by measuring human event-related potentials (ERPs) elicited in response to eight different hues: the four unique hues and the four intermediate hues (orange, lime, teal, and purple). ERPs are waveforms of neural activity that are recorded from the scalp with electrodes and are time-locked to an event, such as a color being shown^32^. We firstly accounted for individual variation in the positions of the unique and intermediate hues by adopting a psychophysical task similar to that used in a prior study^33^. For each of the eight hues, observers selected a hue that was like neither of its neighboring hues on the hue circle (e.g., a red that is neither too orange nor too purple). For each observer (*N* = 23) we then measured the electrophysiological activity elicited in response to their specific unique and intermediate hues. Observers viewed each hue presented in isolation on a neutral background. Observers were required to manually respond to a target hue that varied across blocks. Response trials were excluded from subsequent analysis to avoid the contamination of ERP waveforms from the electrophysiological activity elicited by making a manual response. We had no a priori hypotheses about the exact time that a neural signature of the unique hues would arise during visual processing, and so we analyzed multiple visual ERP components (P1, anterior N1, and posterior P2) that are elicited at various times from color onset.

## Results

**Figure 1:**
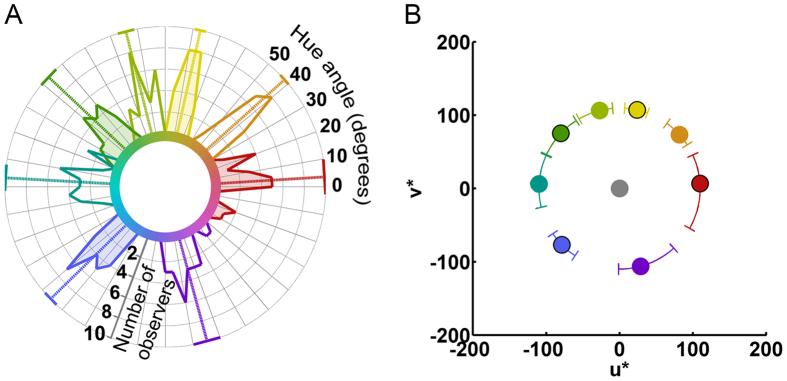
The location and variability of participants’ unique and intermediate hues in a perceptual color space. (**A**) Polar histogram plotting median hue selections for each observer for the four unique hues (shaded) and four intermediate hues (not shaded). Hue is defined by hue angle in CIELUV color space. Colored radial lines represent the mean of these median selections with corresponding 95% confidence intervals as solid lines along the circumference of the plot. (**B**) Mean hue selections for each of the four unique hues (with black border) and the four intermediate hues (without border) defined in a Cartesian plot of CIELUV color space. The axes of the plot define a color according to its redness-greenness (u*) and blueness-yellowness (v*). The circumferential error bars denote the range of median hue selections across observers. The gray circle indicates the chromaticity of the gray background (the white point metameric with CIE Illuminant D65).

Figure 1 provides a summary of the unique and intermediate hue settings: Fig. 1A is a polar histogram of the median unique and intermediate hue settings for all observers; Fig. 1B plots the mean stimulus position of each hue across all observers, as well as the range of hue settings in a perceptual color space (CIELUV). For a figure showing the positions of unique and intermediate hue settings in the physiological MacLeod-Boynton chromaticity diagram^34^, see Supplementary Fig. S4. Each observer’s unique and intermediate hue settings were used to define the stimuli presented to that individual in the ERP task.

For the ERP results we analyzed the peak latencies (ms) and mean amplitudes (μV) of each of the eight hues in three visual ERP components (P1, anterior N1, and posterior P2). A sample of representative ERP waveforms which illustrate these components is given in Supplementary Fig. S1a. The average peak latency and mean amplitude for these components for each hue are presented in Supplementary Table S1.

**Figure 2:**
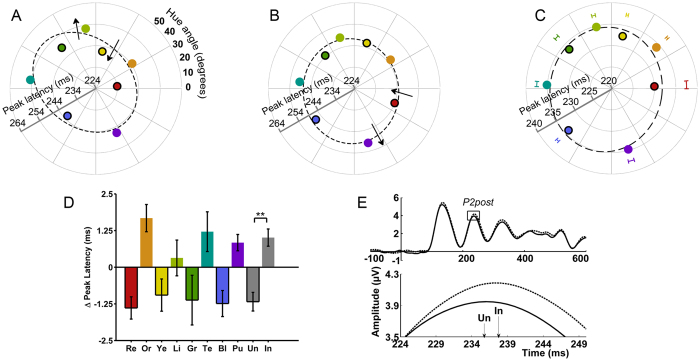
A neural marker of the unique hues in the posterior P2 latencies. (**A**) and (**B**) Neural activity averaged over nine channels located over the extrastriate visual cortex from single observers and depicted as polar plots showing peak latency (*r*) of the posterior P2 as a function of the observer’s median hue selection (*θ*) in CIELUV. The four unique hues of red (Re), yellow (Ye), green (Gr) and blue (Bl) are denoted with solid black borders. The four intermediate hues of orange (Or), lime (Li), teal (Te) and purple (Pu) do not have borders. For each observer, the unique hues fall inside a best-fitting ellipse (dotted black line) showing they all peaked earlier than expected for their location in chromaticity space. Plots have been rescaled for graphical purposes so that the center of each plot starts at 224 ms. Arrows highlight that unique hues fall inside and intermediate hues outside the ellipse. (**C**) Data averaged across all observers (peak latency could not be reliably ascertained for two observers, therefore N = 21), whereby theta represents the mean of the median hue selections, and rho the mean posterior P2 peak latency. Error bars are ± 1 SEM of the median hue selections. (**D**) Group mean residuals of the positions of each hue from the best fitting ellipse for posterior P2 peak latency (bars for unique hues have black borders). The combined mean Unique (Un) and Intermediate (In) residuals are shown in gray. Error bars are ± 1 SEM. (**E**) Averaged ERP waveforms from electrode Oz, a representative midline channel located over the occipital lobe selected here to illustrate the effect (statistical analyses were conducted on waveforms averaged across 9 posterior electrodes). ERP waveforms are averaged for the unique hues (solid line) and the intermediate hues (dotted line). Top of panel **e** depicts mean amplitude across observers from electrode Oz and the posterior P2 component is indicated by a surrounding box. Bottom of **e** shows an enlarged view of the posterior P2 peaks (arrows indicate the peak of the component for unique and intermediate hues).

Our stimuli fall along a circle in the color space that we used to define the colors. However, we observed that the peak latencies and mean amplitudes for individual observers were non-randomly distributed across regions of this color space. For example, Fig. 2A and B shows the mean peak latency of the posterior P2 component as a function of hue (the hue angle in CIELUV color space) for two observers. The data points in each panel form elongated distributions. To account for this individual variability, for each ERP component we fit ellipses to each observer’s peak latency (*r*) and mean amplitude (*r*) as functions of hue angle (*θ*). Ellipses for the two observers shown in Fig. 2A and B are indicated by the dashed lines.

We found the residuals for each data point to the best-fitting ellipse, which is equivalent to the difference in the peak latency or mean amplitude for a particular hue compared to the peak latency or mean amplitude expected from that hue’s position in color space. The sign of each residual (i.e., positive or negative) indicates whether the peak latency or mean amplitude for each hue is smaller or larger than expected for its position in color space, e.g., negative residuals fall inside the ellipse and indicate an earlier peak latency or smaller mean amplitude than expected. This method of fitting ellipses to account for the non-uniform effect of position in color space on a behavioral measure follows that used by refs 35 and 36.

For peak latency and mean amplitude of each component, the ellipse fitting method produced eight residuals for each observer, one for each hue. A series of Friedman tests were conducted on the residuals for peak latency or mean amplitude of each component with Hue (8 levels) as a factor. The crucial result is that for the latency of the posterior P2 component, a Friedman test found a significant main effect of Hue (χ^2^ (7) = 27.5, *p* = 0.0003; α = 0.008 for a 2 × 3 Bonferroni correction for latency and amplitude for 3 ERP components), which was specifically associated with a difference between the grouped unique compared to the grouped intermediate hues. This can be seen in Fig. 2C, which displays mean peak latency of the posterior P2 component, across observers, as a function of hue angle, as well as in Fig. 2D, which presents the group mean residuals of the positions of each hue from the best fitting ellipse. The residuals for all four unique hues are negative (meaning that the posterior P2 occurs earlier than expected), while the residuals for all four intermediate hues are positive. A Wilcoxon test confirmed that the unique hues as a group had significantly earlier posterior P2 peak latency than the intermediate hues (*Z* = −2.9, *p* = 0.004).

This difference in peak latency in the ERP waveforms is presented at a representative electrode site in Fig. 2E. There was also a significant main effect of Hue on the peak latency of P1 (χ^2^ = 16.6, *p* = 0.02). However, the differences amongst hues here were not related to whether or not the hue was unique or not as when we grouped the four unique hues together and compared this to the four grouped intermediate hues, a Wilcoxon test found no significant difference between unique and intermediate hues (*Z* = −1.35, *p* = 0.18; Supplementary Fig. S2a). There was no main effect of Hue on anterior N1 peak latency (Supplementary Fig. S2b). Friedman tests found no significant main effect of Hue on mean amplitude for any component (Supplementary Fig. S2c–e). The effect of unique hues on the posterior P2 peak latency did not depend on our particular analysis using fitted ellipses: a significant main effect of hue was also present in the raw posterior P2 latencies, χ^2^ (7) = 14.2, *p* = 0.048, with a significant difference between unique and intermediate hues, *Z* = −2.8, *p* = 0.005.

## Discussion

The concept of unique hues has a long history and the question of their existence is important for color science and for broader debates in linguistics, philosophy and neuroscience. Despite their importance, neural evidence for the unique hues has not previously been convincingly demonstrated. We report a neural signature of the unique hues that exists 230 ms after a color is presented. The effect was strong: all four unique hues elicited an earlier posterior P2 peak latency than all four intermediate hues. We find no indication of a neural marker for the unique hues in earlier components (i.e., the P1 and anterior N1).

The P2 component is thought to reflect “post-perceptual” processes^37^, coming later than the P1 and early-phase N1 components, which are thought to reflect processes generated by the early visual system^38^. The posterior P2 has been associated with a range of visual cognitive processes including attention^39^, stimulus ambiguity^40^, perceptual learning^41^,^42^, working memory^43^, stimulus detectability^44^,^45^, contour integration^46^ and language processing^47^.

One issue to consider is the role of attention in the difference in latency of the posterior P2 for unique and intermediate hues. Participants were not required to respond on the basis of whether a target was (or was not) a unique hue. Therefore, there was no explicit or implicit requirement to attend more or less to the unique hues compared to the intermediate hues and a strategy such as this would not facilitate performance on the task. However, one could argue that when colors are seen the unique hues garner more attention than the intermediate ones and that the effect in peak latency of the posterior P2 indicates such an attentional effect. However, we find this account unlikely. First, visual attention based on non-spatial features (such as color) will show an ERP activity called selection negativity (SN)^39^,^48^. SN is a negative ERP activity associated with the processing of the attended feature. For color, SN is a strong activity distributed over the occipital region from around 150 ms till around 350 ms if that color is attended. This SN activity, however, was not found in the current study. Second, we find effects in the latency of the posterior P2 (over the occipital area) which, as opposed to the anterior or vertex P2 (over the frontal or central areas), is not commonly associated with attentional effects (e.g., ref. 49). When the posterior P2 effects are related to attention, they are found in the mean amplitude rather than the latency^50^,^51^,^52^.

Our results show different latencies of the posterior P2 for the unique and intermediate hues, and “uniqueness” is one defining characteristic of the distinction. However, there could be an alternative scheme in which the hue categories that the unique hues belong to are advantaged. Hues from red, green, blue and yellow categories could achieve faster latencies because their representations are more accessible than those from other categories, independently of the uniqueness of the stimuli. Accessibility could be determined either linguistically (e.g., if red were faster to name than orange), or non-linguistically (e.g., the category green may have a stronger neural representation than the category teal if green objects occur more frequently than teal objects). We will discuss each of these possibilities in turn.

Though our participants were not required to name the colors to do the task, language may have exerted an influence on our results if either an explicit or implicit linguistic strategy was adopted when memorizing and subsequently identifying the target. Red, green, blue and yellow hues are all named with basic color terms, which are known to all speakers, are monolexemic, and are not subordinate to another color term^12^. There is a known linguistic advantage (e.g., word frequency and response times) for basic over non-basic color terms such as lime or teal^53^. However, two of our intermediate hues, purple and orange also have basic color terms, and studies of color naming show no response time advantage for naming red, yellow, green and blue over colors named with other basic terms^54^,^55^. Therefore, nameability by a basic color term cannot necessarily account for our pattern of results where red, yellow, green and blue and peaked earlier than purple and orange as well as teal and lime.

If a non-linguistic category advantage for red, green, blue and yellow could account for our results, then there would have to be an ease of access advantage for these categories over other frequently used color categories in a task that does not require color naming. Though our participants were not asked to respond as quickly as possible on target trials, we collected behavioral response times to each hue target during the ERP recording session. We found no significant difference in mean response time for red, green, blue and yellow compared to the orange and purple hues, which are also from basic color categories (*Z* = −0.54, *p* = 0.59). Though to our knowledge a non-linguistic category advantage for red, green, blue and yellow has not previously been sought, in our data there is no evidence that red, green, blue and yellow have a stronger non-linguistic categorical representation than other frequently used colors. Therefore, a non-linguistic category advantage cannot obviously account for our results.

A potential advantage for colors from red, green, blue and yellow categories could be linguistic or non-linguistic, but both possibilities rely on our stimuli being close to focal examples of those categories. Focal colors are the best color examples for each category, and are typically defined in a color space that includes variation in luminance and saturation, so are defined separately from the unique hues. For example, focal red is saturated and of a low luminance, while focal yellow is found at a higher luminance^56^. By contrast, unique hues are defined along loci in color space that include a range of saturations and luminances – unique blue is a blue that is neither reddish or greenish but could be dark, light, or of high or low saturation. Our stimuli were defined to be isosaturated and isoluminant (in CIELUV color space) to ensure that we could isolate uniqueness independently of luminance and saturation. Although unique hues are similar in hue to focal red, green, blue and yellow^57^,^58^,^59^, their different lightnesses and saturations distinguish them from focal colors. For example, our unique red was less saturated than focal red, and might even be labeled pink if observers were asked to name it using a single color term. Therefore, even if focal red, green, blue and yellow are at a linguistic or conceptual advantage, that advantage does not necessarily apply to our colors, which were defined according to their uniqueness.

The origin of the unique hues has been mysterious. They are not encoded at an early retino-geniculate level of visual processing, and here we find no significant correlate around the first 100 ms after a color is presented in the early sensory ERP components P1 and early-phase N1. Our results are consistent with a later neural representation. But what could cause the unique hues to receive their uniqueness? The perceptual salience hypothesis has long proposed that the unique hues are hardwired and that confers the cross-cultural consensus in the position of focal colors^13^,^57^. However, this framework has been weakened by the fact that unique hues do not show a privileged position in behavioral response times^31^,^60^, discriminability^35^ but see also ref. 47, consistency^36^ and perceived saturation^59^. Our finding of an effect of uniqueness around 230 ms is neutral with respect to the perceptual salience hypothesis (since the P2 is not a clear marker for perceptual salience), yet the effect does provide evidence that unique hues are not solely a linguistic construct, but that they are represented at some other level.

An alternative but not mutually exclusive account to the perceptual salience hypothesis favors an environmental origin of unique hues, which would become internalized either genetically or ontogenetically. Unique blue and yellow could arise from familiarity and normalization to the color statistics of natural scenes, and particularly daylight illuminants^29^,^61^,^62^,^63^, though there is currently not a good account in this framework for the origin of unique red and green^4^. Some have proposed that reflectance spectra corresponding to unique hues generate more reliable color signals across changing illumination^64^,^65^. Alternatively, the social rather than the physical environment may confer the unique hues’ special status through linguistic and cultural consensus^66^. To distinguish the contributions of neural hardwiring, language and environment to the unique hues, their measurement across cultures, and in prelinguistic infants, using a neural marker such as the one we here present, will be critical.

As well as providing a means of testing alternative accounts about the origin of unique hues, our neural marker could be used to address questions in cognitive science and philosophy that have used color as a key testing ground. For philosophers, our finding provides a potential neural correlate of monoadic qualia^6^,^15^. For cognitive scientists, unique hues can be applied to the Whorfian question of the relationship between language and cognition^9^,^13^: Finding a neural marker of the unique hues across cultures with different color lexicons would be in favor of the alternative universalist position. Having now established a neural marker of the unique hues, it could be used to inform and progress these prominent and unresolved questions in the cognitive sciences.

## Materials and Methods

### Participants

Twenty-three native British English speakers took part (10 male; mean age = 19.7; *SD* = 1.36). Observers were recruited from the University of Sussex. All observers had normal color vision, assessed using the Ishihara test^67^ and the City University Test^68^ presented under natural daylight. Observers were naive to the purpose of the study, provided written informed consent and their time was reimbursed with money or research credits. All experimental protocols were approved by the University of Sussex Sciences and Technology Cross Schools Research Ethics Committee and the methods were carried out in accordance with the approved guidelines.

### Set up

Observers were seated in a dark room, the only source of light was the 22″Diamond Plus CRT monitor (Mitsubishi, Tokyo, Japan), which was used to present the stimuli (color resolution: 8 bits∕channel; spatial resolution: 1024 × 768; refresh rate: 75 Hz) and located 40 cm away from observers. Gamma correction was achieved using a CRS ColorCal (Cambridge Research Systems, Rochester, UK).

### Task 1: Hue settings

#### Stimuli

Stimuli were annuli of 100 equally-sized colored segments with an outer diameter of 22° and inner diameter 14°. Each segment was an isosceles trapezoid with a circular top and base covering an area of 2 × 2°. The segments had a geometric angle of 2.6° of the annulus and between them were 1° gaps. The segments were isoluminant (28 cd/m^2^), isosaturated in the CIELUV chromaticity space (L* = 130; chroma = 110) and always sequentially circumnavigated the CIELUV hue circle in equal-sized steps of a hue angle of 3.6°. The hue circle was randomly rotated on each trial so that the exact chromaticity coordinates of the colors would vary and would not appear in the same location on each trial. The background gray was metameric with D65 and had a luminance of 14 cd/m^2^. The experiment was written in Matlab (The MathWorks Inc., 2012) with the Psychophysics toolbox^69^.

Given that there are known to be small differences in the latencies of different colored stimuli when presented on a CRT monitor^70^, we made careful measurements to show unequivocally that no artifact arising from the temporal characteristics of our display could undermine our conclusions. These measurements and their results are described in the Supplementary Information.

#### Design and procedure

At the start of each block observers were instructed to select a particular hue in comparison to its neighboring hues, for example, “an orange that is neither too red nor too yellow”. On each trial observers selected the specified hue by clicking a segment in the annulus with the mouse cursor. The color terms (red, orange, yellow, yellow-green, green, blue-green, blue, and purple) were the same as those used by^33^. Once selected, a light gray highlighter (35 cd/m^2^ and metameric with D65) was displayed outside the annulus with a gap of 2° to the annulus. An alternative segment could be selected in which case the highlighter moved, or the same segment was tapped again to complete a trial. One hue was measured in each block of 20 trials. There were 16 blocks: the order was randomized so that the first eight and last eight blocks each contained all of the eight hues, and each block was different to the last. If participants forgot the target color, they could hold the spacebar during a trial to temporarily show the instructions.

### Task 2: ERPs

#### Stimuli

Stimuli were the four unique hues (red, yellow, blue, and green) and four intermediate hues (orange, lime, teal, and purple) for each observer. The chromaticity coordinates of the eight hues were each observer’s median hue selections from the hue selection task. Test materials were presented with e-Prime 2 (Psychology Software Tools, Inc.).

#### Design and procedure

At the start of each block one of the eight hues was selected randomly as the target hue. The eight hues were presented centrally as squares (2 × 2°) for 400 ms on each trial, with a randomized interstimulus interval of 1200–1600 ms. In a block, all hues were presented 10 times each in a random order. The observer was asked to make a manual response only to the target hue by pressing the space bar with both hands. The task instructions made no reference to unique or intermediate hues: participants were simply required to respond when the target hue was shown. There were 16 blocks, so each hue was presented a total of 140 times as the distracter and 20 times as the target. Prior to testing, observers completed 40 practice trials which were identical to the main trials but the target was black (metameric with D65 with a luminance of 0.60 cd/m^2^).

#### EEG Recording and Analysis

EEG data was recorded and processed with NeuroScan SynAmps^2^ amplifiers and SCAN 4.3 software (NeuroScan/Compumedics, Inc.) at a digitizing rate of 1,000 Hz. A physical band-pass filter was applied to online recording (0.10–100 Hz). EEG was recorded from 39 electrode sites: FP1, FPz, FP2, AF3, AF4, F7, F3, Fz, F4, F8, FC3, FCz, FC4, T7, C3, Cz, C4, T8, CP3, CPz, CP4, P7, P5, P3, P1, Pz, P2, P4, P6, P8, PO7, PO3, POz, PO4, PO8, O1, Oz, O2, and the observer’s right mastoid using Ag-AgCl electrodes physically referenced to the left mastoid. The EEG activities at the mastoids were averaged off-line and used as the reference. Eye blinks and eye movements were monitored via one bi-polar horizontal electro-oculogram (HEOG) channel located laterally of the canthi and one vertical electro-oculogram (VEOG) channel located above the observer’s left eye. Impedance of each channel was reduced below 5 kΩ prior to data collection. Following EEG recording, a zero phase-shift low-pass filter with amplitude cut-off frequency of 30 Hz and 24 dB/oct roll-off was applied to the data. EEG and HEOG were epoched off-line with a window extending 600 ms after stimulus onset, relative to a 100 ms pre-stimulus baseline. Artifact rejection criteria comprised trials with a voltage exceeding ± 60 μV at any electrode site. ERPs were generated by averaging EEG activities over trials time-locked to stimulus onsets.

#### Statistics and data analysis

The ERP data were analyzed with the standard procedure^32^,^71^: For each ERP component, we selected electrode locations showing maximal activities in the topographic map because these locations were representative of the ERP activity. The ERP components were then quantified as peak latencies and mean amplitudes. For the purpose of latency analysis, each ERP component was identified in each observer’s waveforms. If an observer’s ERP waveform did not show a discernible peak for that component at a selected electrode site, this electrode site was excluded from the analysis of this component to avoid taking unreliable latency measures.

In the current study, P1 had a maximal distribution over occipital and parieto-occipital sites (O1, Oz, O2, PO3, POz, & PO4) around 130 ms after stimulus onset. The anterior N1 was maximal around 136 ms after stimulus onset over frontal and fronto-central sites (AF3, AF4, F3, Fz, F4, FC3, FCz, & FC4). The posterior P2 was maximally distributed over occipital, parieto-occipital, and parietal sites around 230 ms after stimulus onset (O1, Oz, O2, PO3, POz, PO4, P1, Pz, & P2). After the screening procedure, one, three, and two observers were excluded from the analysis of the P1, anterior N1, and posterior P2 components respectively. The average of the peak latencies measured at the selected sites was calculated for each hue and each observer before being statistically analyzed.

To acknowledge the timing difference (latencies) of the same ERP component across hue conditions, mean amplitude was quantified over a 30-ms window centered at the peak latency at each of the selected electrode sites. This method provides a more reliable measure than peak amplitudes^32^. The mean amplitude over the selected sites was calculated for each hue and was analyzed.

## Additional Information

**How to cite this article**: Forder, L. *et al*. A neural signature of the unique hues. *Sci. Rep.* **7**, 42364; doi: 10.1038/srep42364 (2017).

**Publisher's note:** Springer Nature remains neutral with regard to jurisdictional claims in published maps and institutional affiliations.

## Supplementary Material

Supplementary Information

## References

[b1] da Vinci L. A Treatise on Painting. (George Bell & Sons, 1651).

[b2] Pridmore R. W. 14th century example of the four unique hues. Color Res. Appl. 31, 364–365 (2006).

[b3] Hering E. Outlines of a theory of the light sense (Translated by Hurvich L. M. & Jameson D.). (Harvard University Press, 1878).

[b4] Broackes J. Where Do the Unique Hues Come from? Rev. Philos. Psychol. 2, 601–628 (2011).

[b5] Sternheim C. E. & Boynton R. M. Uniqueness of perceived hues investigated with a continuous judgmental technique. J. Exp. Psychol. 72, 770 (1966). 5972019 10.1037/h0023739

[b6] Valberg A. Unique hues: an old problem for a new generation. Vision Res. 41, 1645–1657 (2001). 11348647 10.1016/s0042-6989(01)00041-4

[b7] Hård A. & Sivik L. NCS—Natural Color System: A Swedish Standard for Coloer Notation. Color Res. Appl. 6, 129–138 (1981).

[b8] Mollon J. D. & Jordan G. In John Dalton’s colour vision legacy 381–392 (Taylor & Francis, 1997).

[b9] Gumperz J. J. & Levinson S. C. Rethinking linguistic relativity. (Cambridge University Press, 1996).

[b10] Roberson D., Davies I. & Davidoff J. Color categories are not universal: replications and new evidence from a stone-age culture. J. Exp. Psychol. Gen. 129, 369 (2000). 11006906 10.1037//0096-3445.129.3.369

[b11] Whorf B. L. In Science and linguistics (ed. John Carroll) (MIT Press, 1956).

[b12] Berlin B. & Kay P. Basic color terms: their universality and evolution. (University of California, 1969).

[b13] Kay P. & Regier T. Resolving the question of color naming universals. Proc. Natl. Acad. Sci. 100, 9085–9089 (2003). 12855768 10.1073/pnas.1532837100 PMC166442

[b14] Pinker S. The Language Instinct: The New Science of Language and Mind. (HarperCollins, 1995).

[b15] Byrne A. & Tye M. Qualia ain’t in the head. Noûs 40, 241–255 (2006).

[b16] Campbell N. Why We Should Lower Our Expectations about the Explanatory Gap. Theoria 75, 34–51 (2009).

[b17] Kalderon M. E. Color Pluralism. Philos. Rev. 116, 563–601 (2007).

[b18] MacLaury R. E. Color and cognition in Mesoamerica: Constructing categories as vantages. (University of Texas press, 1997).

[b19] Jameson K. A. & Matthen M. In Color Ontology and Color Science 179–202 (MIT Press, 2010).

[b20] Stoughton, C. M. & Conway B. R. Neural basis for unique hues. Curr. Biol. 18, R698–R699 (2008). 18727902 10.1016/j.cub.2008.06.018

[b21] De Valois R. L., Abramov I. & Jacobs G. H. Analysis of Response Patterns of LGN Cells. JOSA 56, 966–977 (1966). 10.1364/josa.56.000966 4959282

[b22] Jameson K. & D’Andrade R. In Color Categories in Thought and Language 295–319 (Cambridge University Press, 1997).

[b23] Mollon J. D. & Cavonius C. R. In Colour vision deficiencies VIII 473–483 (Springer, 1987).

[b24] De Valois R. L. & De Valois K. K. A Multi-Stage Color Model. Vision Res. 33, 1053–1065 (1993). 8506645 10.1016/0042-6989(93)90240-w

[b25] Wuerger S. M., Atkinson P. & Cropper S. The cone inputs to the unique-hue mechanisms. Vision Res. 45, 3210–3223 (2005). 16087209 10.1016/j.visres.2005.06.016

[b26] Mollon J. D. A neural basis for unique hues? Curr. Biol. 19, R441–R442 (2009). 19515347 10.1016/j.cub.2009.05.008

[b27] Bohon K. S., Hermann K. L., Hansen T. & Conway B. R. Representation of Perceptual Color Space in Macaque Posterior Inferior Temporal Cortex (the V4 Complex). eNeuro 3, (2016). 10.1523/ENEURO.0039-16.2016 PMC5002982 27595132

[b28] Bosten J. M. & Boehm A. E. Empirical evidence for unique hues? J. Opt. Soc. Am. A 31, A385 (2014). 10.1364/JOSAA.31.00A385 24695198

[b29] Mollon J. Monge: the verriest lecture, Lyon, July 2005. Vis. Neurosci. 23, 297–309 (2006). 16961961 10.1017/S0952523806233479

[b30] Saunders B. A. C. & van Brakel J. Are there nontrivial constraints on color categorization? Behav. Brain Sci. 20, 167–228 (1997). 10096997

[b31] Wool L. E. . Salience of unique hues and implications for color theory. J. Vis. 15, 1–11 (2015). 10.1167/15.2.10 PMC4319534 25761328

[b32] Luck S. J. An Introduction to the Event-Related Potential Technique. (MIT Press, 2005).

[b33] Malkoc G., Kay P. & Webster M. A. Variations in normal color vision. IV. Binary hues and hue scaling. JOSA A 22, 2154–2168 (2005). 16277285 10.1364/josaa.22.002154

[b34] MacLeod D. I. & Boynton R. M. Chromaticity diagram showing cone excitation by stimuli of equal luminance. JOSA 69, 1183–1186 (1979). 10.1364/josa.69.001183 490231

[b35] Witzel C. & Gegenfurtner K. R. Categorical sensitivity to color differences. J. Vis. 13, 1–33 (2013). 10.1167/13.7.1 23732118

[b36] Bosten J. M. & Lawrance-Owen A. J. No difference in variability of unique hue selections and binary hue selections. J. Opt. Soc. Am. A 31, A357 (2014). 10.1364/JOSAA.31.00A357 24695194

[b37] Patel S. H. & Azzam P. N. Characterization of N200 and P300: selected studies of the event-related potential. Int J Med Sci 2, 147–54 (2005). 16239953 10.7150/ijms.2.147 PMC1252727

[b38] Di Russo F., Martínez A., Sereno M. I., Pitzalis S. & Hillyard S. A. Cortical sources of the early components of the visual evoked potential. Hum. Brain Mapp. 15, 95–111 (2002). 11835601 10.1002/hbm.10010 PMC6871868

[b39] Anllo-Vento L., Luck S. J. & Hillyard S. A. Spatio-temporal dynamics of attention to color: evidence from human electrophysiology. Hum. Brain Mapp. 6, 216–238 (1998). 9704262 10.1002/(SICI)1097-0193(1998)6:4<216::AID-HBM3>3.0.CO;2-6 PMC6873357

[b40] Latinus M. & Taylor M. J. Face processing stages: Impact of difficulty and the separation of effects. Brain Res. 1123, 179–187 (2006). 17054923 10.1016/j.brainres.2006.09.031

[b41] Qu Z., Song Y. & Ding Y. ERP evidence for distinct mechanisms of fast and slow visual perceptual learning. Neuropsychologia 48, 1869–1874 (2010). 20080117 10.1016/j.neuropsychologia.2010.01.008

[b42] Song Y. . Neural correlates of short-term perceptual learning in orientation discrimination indexed by event-related potentials. Chin. Sci. Bull. 52, 352–357 (2007).

[b43] Lefebvre C. D., Marchand Y., Eskes G. A. & Connolly J. F. Assessment of working memory abilities using an event-related brain potential (ERP)-compatible digit span backward task. Clin. Neurophysiol. 116, 1665–1680 (2005). 15908268 10.1016/j.clinph.2005.03.015

[b44] Kotsoni E., Csibra G., Mareschal D. & Johnson M. H. Electrophysiological correlates of common-onset visual masking. Neuropsychologia 45, 2285–2293 (2007). 17452044 10.1016/j.neuropsychologia.2007.02.023

[b45] Straube S. & Fahle M. The electrophysiological correlate of saliency: Evidence from a figure-detection task. Brain Res. 1307, 89–102 (2010). 19854163 10.1016/j.brainres.2009.10.043

[b46] Machilsen B., Novitskiy N., Vancleef K. & Wagemans J. Context Modulates the ERP Signature of Contour Integration. PLoS One 6, e25151 (2011). 21949875 10.1371/journal.pone.0025151 PMC3176325

[b47] Kellenbach M. L., Wijers A. A., Hovius M., Mulder J. & Mulder G. Neural differentiation of lexico-syntactic categories or semantic features? Event-related potential evidence for both. J. Cogn. Neurosci. 14, 561–577 (2002). 12126498 10.1162/08989290260045819

[b48] Keil A. & Müller M. M. Feature selection in the human brain: Electrophysiological correlates of sensory enhancement and feature integration. Brain Res. 1313, 172–184 (2010). 20005214 10.1016/j.brainres.2009.12.006 PMC3664361

[b49] Clark V. P. & Hillyard S. A. Spatial selective attention affects early extrastriate but not striate components of the visual evoked potential. J. Cogn. Neurosci. 8, 387–402 (1996). 23961943 10.1162/jocn.1996.8.5.387

[b50] Johannes S., Münte T. F., Heinze H. J. & Mangun G. R. Luminance and spatial attention effects on early visual processing. Cogn. Brain Res. 2, 189–205 (1995). 10.1016/0926-6410(95)90008-x 7580401

[b51] Luck S. J., Heinze H. J., Mangun G. R. & Hillyard S. A. Visual event-related potentials index focused attention within bilateral stimulus arrays. II. Functional dissociation of P1 and N1 components. Electroencephalogr. Clin. Neurophysiol. 75, 528–542 (1990). 1693897 10.1016/0013-4694(90)90139-b

[b52] Talsma D. & Kok A. Intermodal spatial attention differs between vision and audition: An event-related potential analysis. Psychophysiology 39, 689–706 (2002). 12462498

[b53] Davies I. R. & Corbett G. G. A cross-cultural study of colour grouping: Evidence for weak linguistic relativity. Br. J. Psychol. 88, 493–517 (1997). 9290238 10.1111/j.2044-8295.1997.tb02653.x

[b54] Boynton R. M. & Olson C. X. Locating basic colors in the OSA space. Color Res. Appl. 12, 94–105 (1987).

[b55] Boynton R. M. & Olson C. X. Salience of chromatic basic color terms confirmed by three measures. Vision Res. 30, 1311–1317 (1990). 2219747 10.1016/0042-6989(90)90005-6

[b56] Sturges J. & Whitfield T. W. Locating basic colours in the Munsell space. Color Res. Appl. 20, 364–376 (1995).

[b57] Kuehni R. G. Focal Color Variability and Unique Hue Stimulus Variability. J. Cogn. Cult. 5, 409–426 (2005).

[b58] Miyahara E. Focal colors and unique hues. Percept. Mot. Skills 97, 1038–1042 (2003). 15002843 10.2466/pms.2003.97.3f.1038 PMC1404500

[b59] Witzel C. & Franklin A. Do focal colors look particularly ‘colorful’? J Opt Soc Am A 31, A1–A10 (2014). 10.1364/JOSAA.31.00A365 24695195

[b60] Lindsey D. T. . Color Channels, Not Color Appearance or Color Categories, Guide Visual Search for Desaturated Color Targets. Psychol. Sci. 21, 1208–1214 (2010). 20713637 10.1177/0956797610379861 PMC3050514

[b61] Welbourne L. E., Morland A. B. & Wade A. R. Human colour perception changes between seasons. Curr. Biol. 25, R635–R653 (2015). 10.1016/j.cub.2015.06.030 26241135

[b62] Lafer-Sousa R., Liu Y. O., Lafer-Sousa L., Wiest M. C. & Conway B. R. Color tuning in alert macaque V1 assessed with fMRI and single-unit recording shows a bias toward daylight colors. JOSA A 29, 657–670 (2012). 22561924 10.1364/JOSAA.29.000657

[b63] Bosten J. M., Beer R. D. & MacLeod D. I. A. What is white? J. Vis. 15, 5 (2015). 10.1167/15.16.5 PMC4675320 26641948

[b64] Philipona D. L. & O’regan J. K. Color naming, unique hues, and hue cancellation predicted from singularities in reflection properties. Vis. Neurosci. 23, 331–339 (2006). 16961964 10.1017/S0952523806233182

[b65] Witzel C., Cinotti F. & O’Regan K. What determines the relationship between color naming, unique hues, and sensory singularities: Illuminations, surfaces, or photoreceptors? J. Vis. 15, 1–32 (2015). 10.1167/15.8.19 26114682

[b66] Webster M. A. . Variations in normal color vision. III. Unique hues in Indian and United States observers. JOSA A 19, 1951–1962 (2002). 12365615 10.1364/josaa.19.001951

[b67] Ishihara S. Ishihara test for colour-blindness. (Kanehara & Co. Ltd, 1987).

[b68] Fletcher R. The City University Colour Vision Test. (Keeler, 1980).

[b69] Brainard D. H. The psychophysics toolbox. Spat. Vis. 10, 433–436 (1997). 9176952

[b70] Vingrys A. J. & King-Smith P. E. Factors in Using Color Video Monitors for Assessment of Visual Thresholds. Color Res. Appl. 11, S57–S62 (1986).

[b71] Rugg M. D. & Coles M. G. H. Electrophysiology of Mind: Event-related Brain Potentials and Cognition. (OUP Oxford, 1996).

