# Supplementary material for: A neural signature of the unique hues
Source: Sci Rep. 2017 Feb 10;7:42364. doi: 10.1038/srep42364 (PMC5301231; doi:10.1038/srep42364)
Supplement: Supplementary Information [file srep42364-s1.pdf]

Supplementary Information

A neural signature of the unique hues

Lewis Forder[a1†]

Jenny Bosten[b]

Xun He[c]

Anna Franklin[a1]

[a]The Sussex Colour Group, University of Sussex, Falmer, BN1 9QH, United Kingdom;
[b]School of Psychology, University of Sussex, Falmer, BN1 9QH, United Kingdom; and
[c]Cognition and Cognitive Neuroscience Research Centre, Department of Psychology, Bournemouth University, Poole, BH12 5BB, UK

[1]To whom correspondence may be addressed: forder@wisc.edu or anna.franklin@sussex.ac.uk

[†]Present address: University of Wisconsin-Madison, Madison, WI 53706, USA

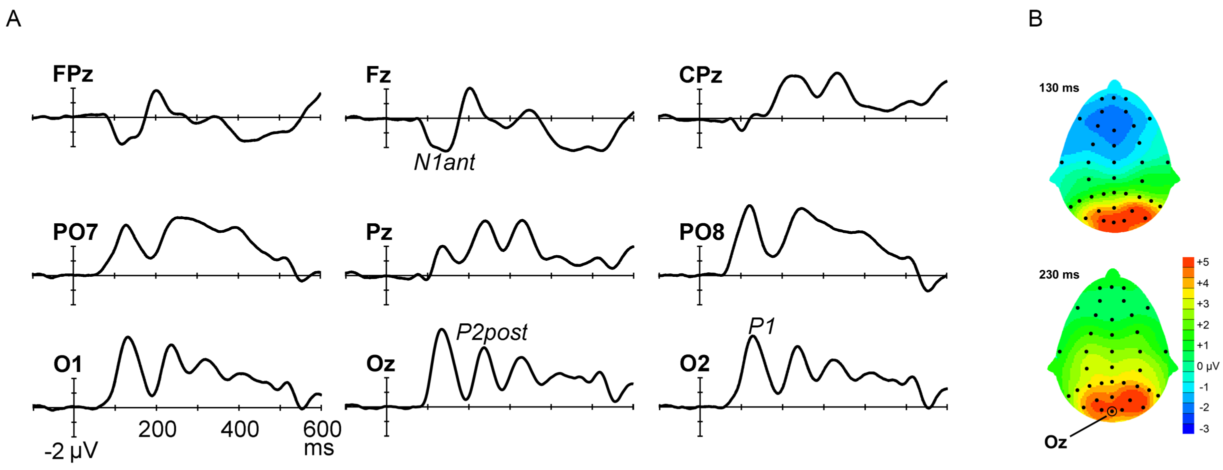

Supplementary Figure S1. Representative ERP waveforms and topographic maps. (**a**) Grand averaged ERP waveforms at nine electrode locations, averaged for all participants (N = 23). Due to the similarity in waveforms across the eight hues and for graphical purposes the waveforms have been averaged across the eight hues to produce a single plot for each electrode. The electrode location (e.g., FPz) is specified at the top of each plot above the *y*-axis. The three ERP components (e.g., P1) are each specified in italics on a single plot. *N1ant:* The anterior N1 component. *P2post:* The posterior P2 component. (**b**) Topographic maps depicting the location of maximum amplitude (µV) for the three ERP components (top figure: P1 and anterior N1; bottom figure: Posterior P2). The position of electrode Oz is located on this figure as Oz is chosen in figure 2E of the main text to illustrate the effect of unique hues in the posterior P2.

Supplementary Table S1. Mean peak latencies and amplitudes for the three ERP components for the four unique hues (white background) and four intermediate hues (gray background). Unique: Mean scores averaged for the four unique hues. Inter: Mean scores averaged for the four intermediate hues.

	**P1**		**Anterior N1**		**Posterior P2**	
	Latency (ms) ± SEM	Amplitude (μV) ± SEM	Latency (ms) ± SEM	Amplitude (μV) ± SEM	Latency (ms) ± SEM	Amplitude (μV) ± SEM
Red	130.86 ± 3.76	5.11 ± 0.73	137.36 ± 5.01	-3.08 ± 0.45	229.64 ± 3.91	4.73 ± 0.49
Orange	130.57 ± 3.51	5.24 ± 0.67	138.41 ± 4.96	-3.13 ± 0.46	233.81 ± 4.20	5.24 ± 0.37
Yellow	128.67 ± 4.13	5.09 ± 0.76	135.24 ± 4.53	-3.08 ± 0.46	232.09 ± 4.28	4.61 ± 0.44
Lime	130.89 ± 3.51	4.90 ±0.71	136.26 ± 4.92	-3.05 ± 0.41	234.40 ± 4.24	4.83 ± 0.41
Green	129.58 ± 3.94	4.83 ±0.71	134.48 ± 4.98	-2.92 ± 0.37	233.10 ± 4.98	5.05 ± 0.54
Teal	129.69 ± 4.33	5.26 ± 0.75	136.35 ± 4.85	-2.52 ± 0.46	234.77 ± 3.90	5.20 ± 0.46
Blue	130.35 ± 3.98	4.76 ± 0.78	138.81 ± 4.86	-2.74 ± 0.52	233.68 ± 4.81	4.61 ± 0.48
Purple	132.69 ± 3.30	5.09 ± 0.75	133.51 ± 4.26	-2.33 ± 0.38	234.38 ± 4.17	4.42 ± 0.47
Unique	129.86 ± 3.87	4.95 ± 0.75	136.47 ± 4.64	-2.96 ± 0.41	232.13 ± 4.28	4.75 ± 0.43
Inter	130.96 ± 3.41	5.12 ± 0.71	136.07 ± 4.45	-2.76 ± 0.38	234.34 ± 4.02	4.93 ± 0.39

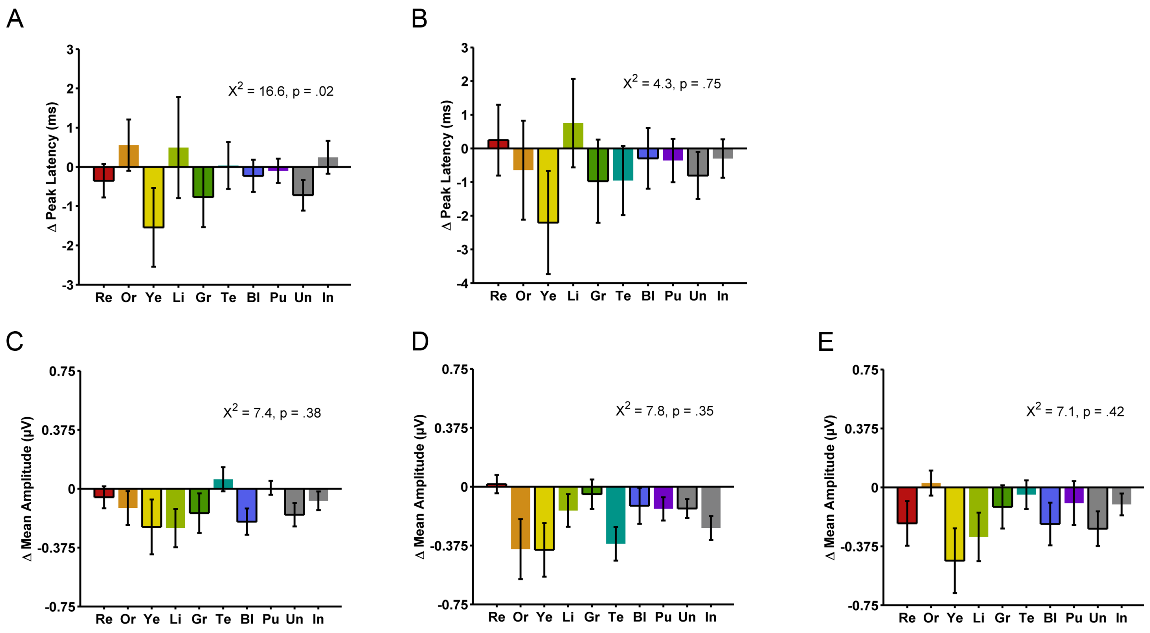

Supplementary Figure S2. Analysis of ERP components. Data corresponds to group mean residuals of the positions of each hue from the best fitting ellipse applied to each observer's data individually. The four unique hues of red (Re), yellow (Ye), green (Gr) and blue (Bl) are denoted with solid black borders. The four intermediate hues of orange (Or), lime (Li), teal (Te) and purple (Pu) do not have borders. The combined mean for each of these groups (Unique: Un; Intermediate: In) are likewise shown and in gray. (**a**) P1 peak latency. (**b**) Anterior N1 peak latency. (**c**) P1 mean amplitude. (**d**) Anterior N1 mean amplitude. (**e**) Posterior P2 mean amplitude. The predominance of negative averaged residuals highlights a slight negative skew in the distribution of the raw averaged ERP data means. Note that for the analysis of ERP mean amplitude and peak latency we used statistical methods that do not make the parametric assumption of normality. Error bars are ± 1 SEM.

Measuring the onset latencies of our stimuli

We found that unique hues had significantly earlier peak latencies than intermediate hues in the posterior P2 component. Since the onset latencies of different colored stimuli presented on CRT monitors are known to vary, we made careful measurements of the presentation latencies of our stimuli using a photodiode connected to an EEG system running at a high (4,000 Hz) sampling rate.

We measured the onset latencies of the four unique and four intermediate hues used in the EEG task. We used the same 22" Diamond Plus CRT monitor (75 Hz refresh rate) that was

used for Tasks 1 and 2 described above. Recall that each participant was presented with their individual unique and intermediate hues following psychophysical measurement (Task 1). Consequently, we did not have a single set of color coordinates for the eight hues, and so opted to use the color coordinates from an observer whose results showed the typical pattern (this observer's results are shown in Fig. 2a in the main article). We also measured the onset latencies of the three CRT primaries at maximum intensity because these would likely highlight the largest differences in onset latency that may occur between different hues. We used the same in-house program that we used in the EEG task to present these 11 hues, but with the inter-frame stimulus darkened to allow accurate measurement of the times of stimulus onset. While the measurements were being made the only source of light was the CRT monitor itself.

The eight hues were each presented individually about 60 times in a randomized order across several blocks, using the same experimental script that we used to conduct Task 2. The stimuli were presented on a black background so that the voltage elicited when a particular hue was displayed could be accurately identified. The photodiode was attached to the center of the CRT monitor. The three primaries were subsequently measured in the same fashion.

The photodiode was an XE-258 (ANT Neuro, Enschede, The Netherlands), with a wavelength sensitivity range of 400 – 1100 nm and rise time of less than 100μs. The photodiode was connected to a 64 channel ANT Neuro amplifier (Enschede, The Netherlands) and output was digitized at a sampling rate of 4,000 Hz.

Supplementary Fig. S3a shows the normalized voltage of each of the four unique and four intermediate hues corresponding to one instance of vertical refresh and averaged over multiple measurements. The average normalized voltage is plotted on the y-axis and time (ms) on the y-axis. Supplementary Fig. S3b shows the same for the monitor primaries. For each of these eleven hues, we calculated a 50% area latency measure (ms). This corresponds to the point at which the area under the curve for one frame is equal on both sides. An independent measures ANOVA found that the eight experimental hues (four unique and four intermediate) significantly varied at the time point that they reached 50% area latency, $F(7,337) = 8.2$, $p < .001$. This can be seen in the averages presented in Supplementary Fig. S3c, which shows a tendency for reddish colors to reach 50% area latency slower than bluish colors. This is confirmed in the observations we made of the monitor primaries presented in Supplementary Fig. S3b. Critically, when we grouped the unique hues together and compared their latencies to those of the intermediate hues, we found no significant difference ($t(343) = -1.2$, $p = .233$).

In summary, we found significant differences in the times that different hues reach 50% area latency. The blue primary reached this point fastest and the red primary slowest. However, the pattern we observe for onset latency for the experimental eight hues presented in Supplementary Fig. S3c does not match the pattern we report in the posterior P2 peak latency. Specifically, here the photodiode measurements do not show that the unique hues reach onset latency significantly faster than intermediate hues. Moreover, the size of the difference in onset latency between these grouped unique versus grouped intermediate hues is less than 0.02 ms. The effect we report in the posterior P2 peak latency is more than 100 times larger than this. Our measurements of stimulus onset latencies show unequivocally that our results do not arise from differences in the latencies of presentation of different colors on the CRT monitor.

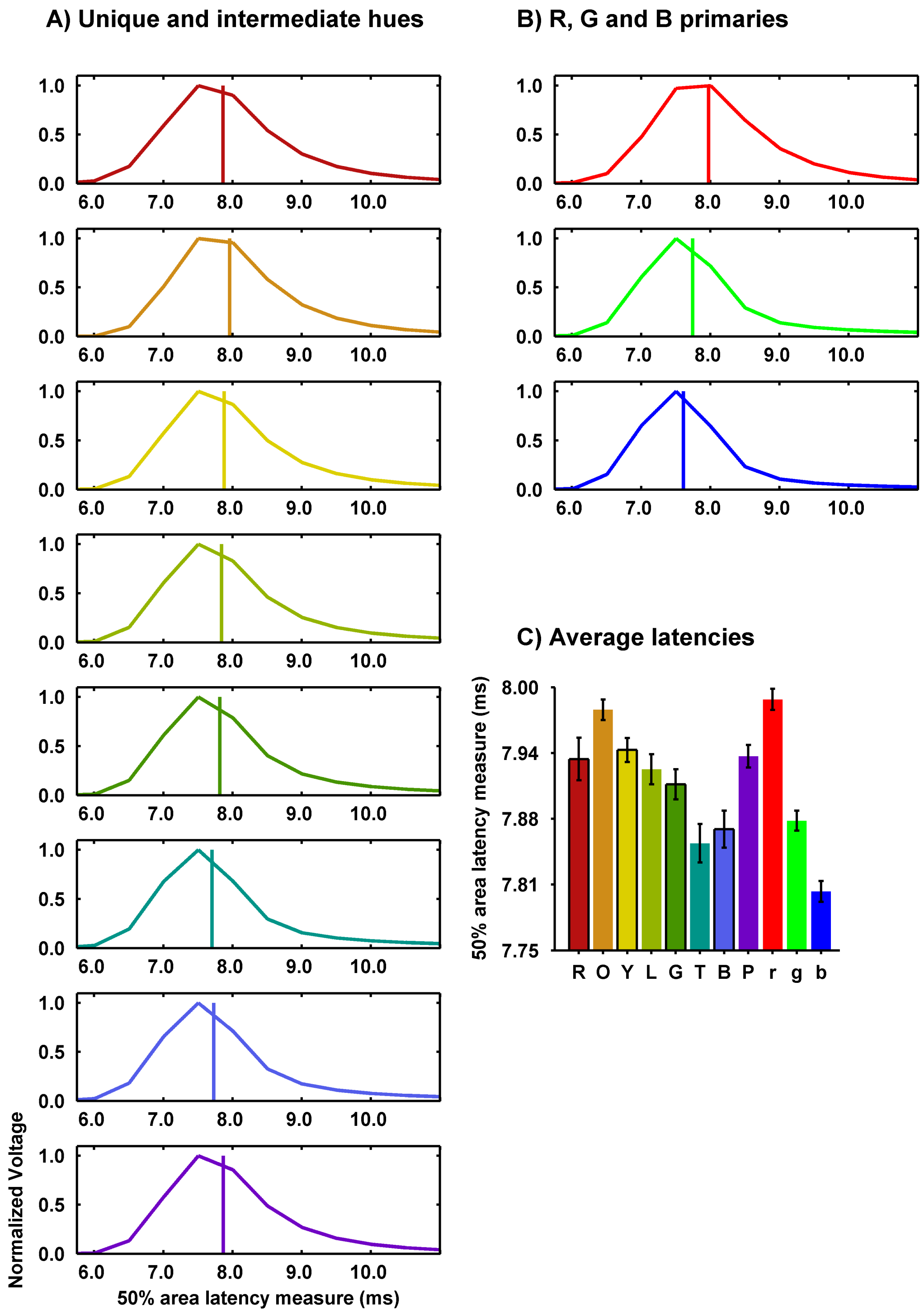

Supplementary Figure S3. Normalized voltage of the onset latency for the eight hues (four unique and four intermediate) used in the EEG task as well as the three monitor RGB primaries. Each waveform is the average taken across multiple measurements and corresponds to the first instance of vertical refresh of the CRT monitor used in Tasks 1 and 2. The vertical line represents the time point (ms) that the waveform is at 50% area latency. (**a**) The four unique and four intermediate. From top to bottom the figure displays unique red,

orange, unique yellow, lime, unique green, teal, unique blue, and purple. (**b**) The three RGB primaries. (**c**) The mean time (ms) that each of the eleven hues reached 50% area latency. Upper case letters correspond to eight the experimental hues: R = red, O = orange, Y = yellow, L = lime, G = green, T = teal, B = blue, P = purple. The unique hues are outlined in black. Lower case letters correspond to the three primaries: r = red primary, g = green primary, b = blue primary. Error bars represent ± 1 SEM. All bars and waveforms are colored accordingly.

Stimuli in a physiological color space

We chose our stimuli along an isosaturated circle in the CIELUV color space. It is important to show that when the stimuli are expressed in a physiologically relevant color space, the unique and intermediate hues are not at saturation extremes. If the unique hues were at more saturated chromaticities than the intermediate hues in a physiologically relevant color space, then it is possible that known low-level color channels could account for the priority for unique hues that we have observed in ERPs. A similar argument was made by Mollon[1] in response to a paper by Stoughton and Conway[2]. In Supplementary Fig. S4 we plot our stimuli (for all participants) in the physiologically relevant MacLeod-Boynton chromaticity diagram[3]. The figure shows that our stimuli fall along an ellipse in the MacLeod-Boynton chromaticity diagram. Though unique red is positioned at a maximum for increments in L/(L+M) and unique yellow at a maximum for decrements in S/(L+M), it is purple that is at the maximum for increments in S/(L+M) and teal at the maximum for decrements in L/(L+M). Unique green in particular is not near a saturation maximum either for S/(L+M) or for L/(L+M). The relative activations that our stimuli induce in the retino-geniculate color mechanisms represented in the MacLeod-Boynton chromaticity diagram therefore cannot account for the neural signature of the unique hues that we have observed.

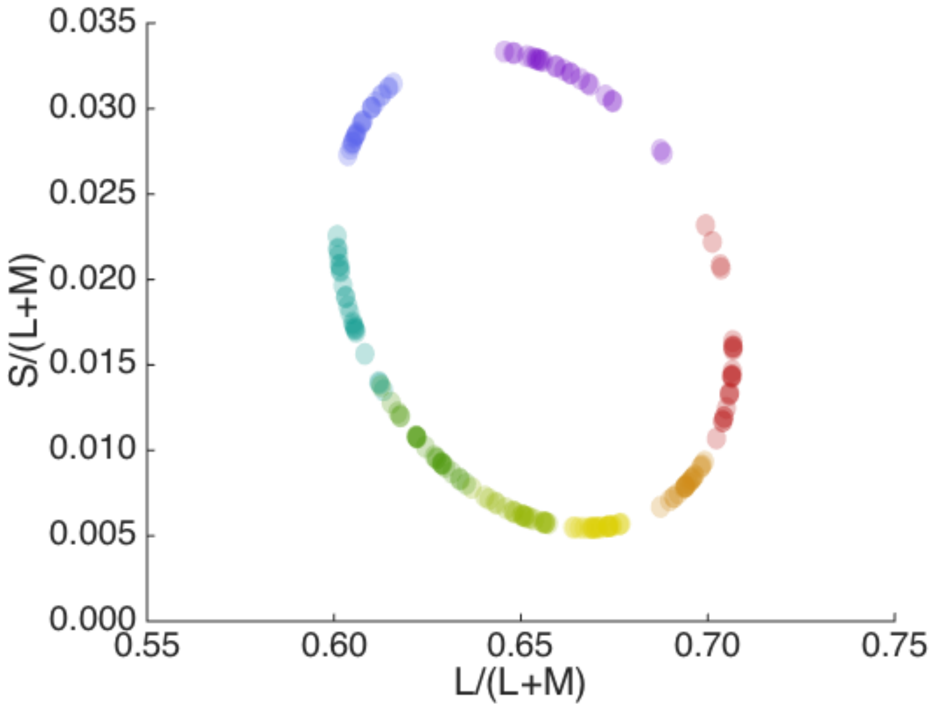

Supplementary Figure S4. Stimuli for all participants plotted in the MacLeod-Boynton chromaticity diagram[3].

References:

1. Mollon, J. D. A neural basis for unique hues? *Curr. Biol.* **19,** R441–R442 (2009).
2. Stoughton, C., M. & Conway, B. R. Neural basis for unique hues. *Curr. Biol.* **18,** R698–R699 (2008).
3. MacLeod, D. I. & Boynton, R. M. Chromaticity diagram showing cone excitation by stimuli of equal luminance. *JOSA* **69,** 1183–1186 (1979).
